# Supplementary material for: Floquet parity-time symmetry in integrated photonics
Source: Nat Commun. 2024 Jan 31;15:946. doi: 10.1038/s41467-024-45226-x (PMC10830577; doi:10.1038/s41467-024-45226-x)
Supplement: Supplementary file 1 — Supplementary Information [file 41467_2024_45226_MOESM1_ESM.pdf]

Supplementary Information: Floquet Parity-Time Symmetry in Integrated Photonics

Weijie Liu[1,*], Quancheng Liu[2,*], Xiang Ni[3,4*], Yuechen Jia[1], Klaus Ziegler[5], Andrea Alù[4,6,†], and Feng Chen[1,†]

[1]School of Physics, State Key Laboratory of Crystal Materials, Shandong University, Jinan, 250100, China

[2]Department of Physics, Institute of Nanotechnology and Advanced Materials, Bar-Ilan University, Ramat-Gan 52900, Israel

[3]School of Physics and Electronics, Central South University, Changsha, Hunan, 410083, China

[4]Photonics Initiative, Advanced Science Research Center, City University of New York, New York, NY 10031, USA

[5]Institut für Physik, Universität Augsburg, D-86135 Augsburg, Germany

[6]Physics Program, Graduate Center, City University of New York, New York, NY 10016, USA

[*]These authors contributed equally to this work.

[†]Corresponding authors: aalu@gc.cuny.edu; drfchen@sdu.edu.cn

S1. Experiment for femtosecond laser writing of waveguides

Fig. S1(a) shows the experimental setup for fabrication of the waveguides, using femtosecond laser writing. The details for the fabrication can be seen in Method. In this work, the light intensity distribution of the structure at different propagation lengths is required. The method we take is to fabricate structures of different lengths, as shown in Fig. S1(b). The output of the structure is located on the back surface of the substrate, and the input waveguide of the structure is guided by a single waveguide to the front surface of the substrate.

The experiments are performed by an end-face coupling system. A He-Ne laser (at 633 nm) is injected into the selected waveguide of the system by a microscope objective (20×/0.4 N.A.) to excite waveguide modes. And the output intensity distribution (beam evolution) is measured (monitored) at the output facet using a CCD camera by another

microscope objective (20×/0.4 N.A.). The morphology of structural end facets is measured by a metalloscope (Axio Imager, Carl Zeiss).

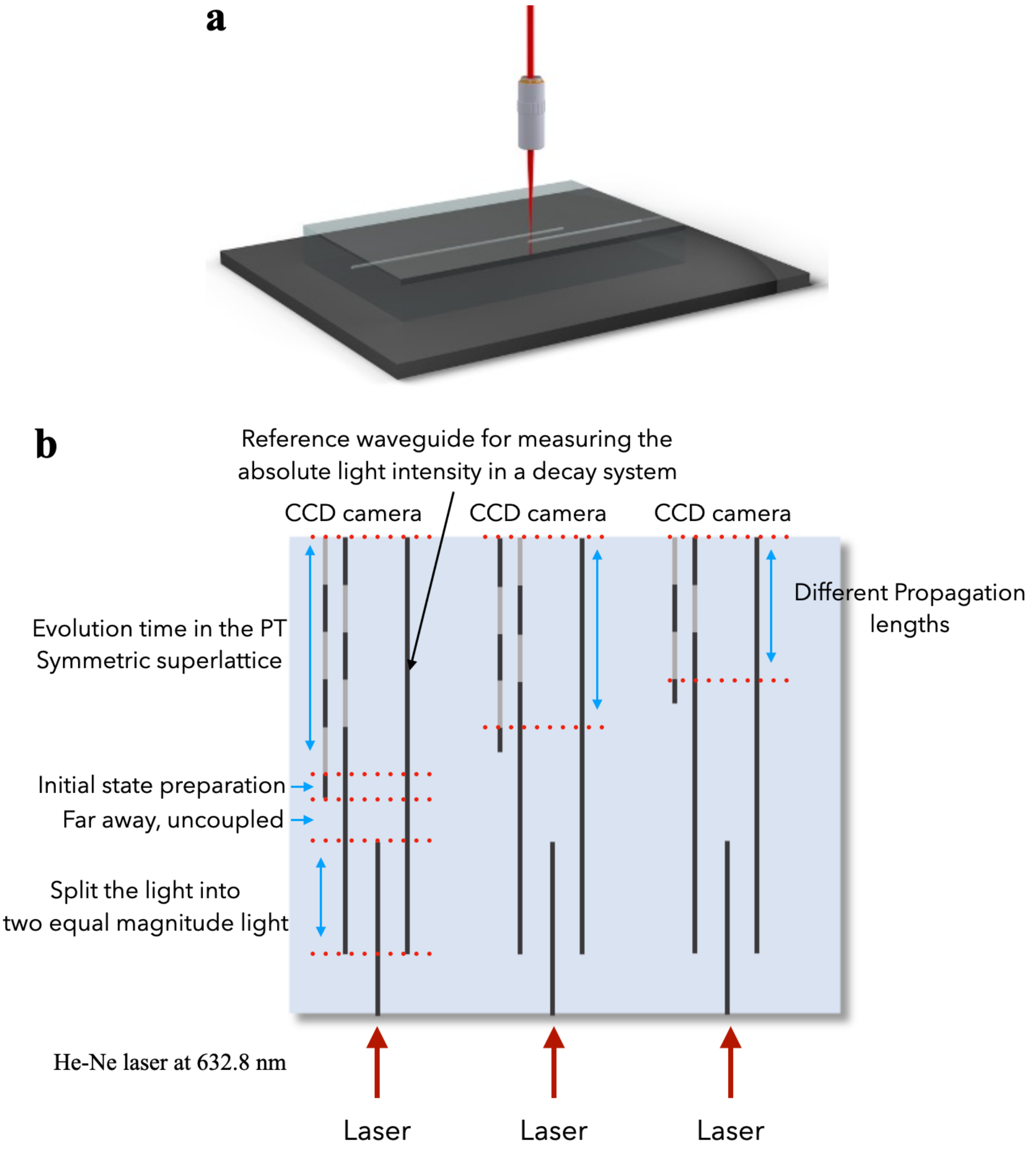

Fig. S1. Experimental setups. a, Schematic of femtosecond laser fabrication of waveguide structures. **b,** Schematic of fabricated structures of different lengths for investigation. Here the black line denotes the straight waveguide and the gray waveguide denotes the curved waveguide.

S2. Preparation of the initial states for the Floquet PT symmetric system and the loss rate measurement

We first measure the coupling magnitude between two straight waveguides, which is crucial for the preparation of the magnetization and decay initial states. We excite two coupled waveguides with a distance of $d = 10$ µm from the left one with a 633 nm He-Ne laser. Then we measure the light intensity on the right waveguide for different propagation lengths. As shown in Fig. **S2(a)**, the light intensity on the right waveguide can be described by the function $I = I_0 = \sin^2(\kappa l)$, where κ is the coupling magnitude between the two waveguides we plan to measure, and l is the light propagation length in the z direction. We experimentally measure 19 light intensities in the distance of L = 10 mm and then fit the experimental data with the function. It is shown that the coupling between the two waveguides $\kappa = 314$ m^{-1}. Hence the oscillation period is $T_R = 10$ mm. For the preparation of the amplification and suppression initial states, the propagation distance in the waveguide array is $L = T_R/4 = 2.5$ mm, where the $|\xi_{am}\rangle$ is realized by injecting the laser from the right waveguide and the $|\xi_{su}\rangle$ is achieved by exciting the array from the left waveguide. The measurement of light intensity for these two initial states is shown in Fig. **S2a**, where the light intensities are equal on both waveguides.

In Fig. **S2(b)**, we measure the effective light loss rate Γ_{sw} for the curved waveguide. We use a straight waveguide of a total length $L_0 = 30$ mm as the reference. We excite this waveguide from the left side and measure the light intensity on the right side, where the light intensity I_0 is recorded[2]. For the sin curved waveguide in separate lines, we also keep the total length $L_0 = 30$ mm. The sin curved waveguide is far away from the straight waveguide. Hence the coupling between them can be negligible. We also excite the waveguide portions from the left side and measure on the right for different ms. The light intensity ratio between the straight waveguide and curved waveguide can be used to calculate the decay rate Γ_{sw}, i.e., $\exp(-\Gamma_{sw} l) = I/I_0$. As shown in the Figure, the measured loss rate $\Gamma_{sw} = 100.4$ m^{-1}.

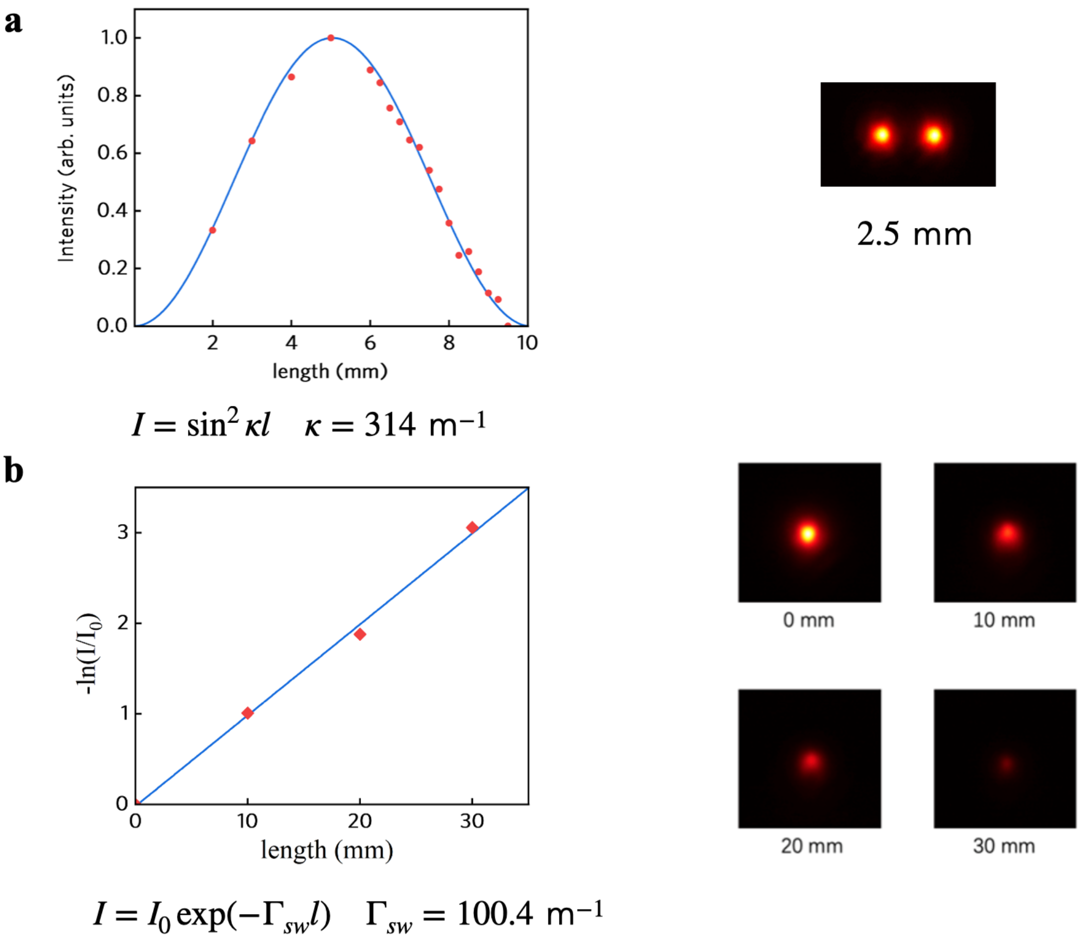

Fig. S2. Measuring coupling strength between two waveguides and loss magnitude for the single twisted waveguide. a, Measured light intensity on the right waveguide versus the propagation length (red dots). The blue line is the fitting curve for $\kappa = 314$ m^{-1}. The initial states for the PT superlattice are prepared with $L = 2.5$ mm. As shown in the right side of the Fig, S2(a) the light intensity from the two waveguides is equal. **b,** Fitting the effective loss magnitude Γ_{sw} for the sin curved waveguide.

S3. Analytical derivation of the Floquet PT symmetric system

In this section, we present the derivations for the Floquet PT symmetric system. We calculate the Floquet propagator for the time-dependent Hamiltonian with up and down PT Hamiltonians (Eq. (1) in the main text) for the special case with analytically solved solutions. Let us denote the initial state is $|\psi_0\rangle$. In general, the time evolution of the system driven by the time-dependent Hamiltonian $H(t)$ can be formally written as:

$$\left|\psi(t)\right\rangle = \mathcal{T}\, e^{-i\int_0^t H(t)dt}\left|\psi_0\right\rangle, \quad \text{(S1)}$$

where $\mathcal{T}$ executes the time ordering and $H(t)$ is periodic with $H(t+T)$ and composed of the up and down PT symmetric Hamiltonians $H_{pt}^{\uparrow}$ and $H_{pt}^{\downarrow}$. Following the main text, we denote the evolution time by $H_{pt}^{\uparrow}$ is $\tau_{\uparrow}$ and the evolution time by $H_{pt}^{\downarrow}$ is $\tau_{\downarrow}$, then the driven period is $T = \tau_{\uparrow} + \tau_{\downarrow}$. Then the evolution of the Floquet PT symmetric system is:

$$|\psi(nT)\rangle = [e^{-iH_{pt}^{\downarrow}\tau_{\downarrow}}\, e^{-iH_{pt}^{\uparrow}\tau_{\uparrow}}]^n|\psi_0\rangle = \left(U^{\uparrow,\downarrow}\right)^n|\psi_0\rangle. \quad \text{(S2)}$$

To investigate the property of the Floquet PT symmetric system, we need to solve the propagator $U^{\uparrow,\downarrow}$ for one period time T. Here we expand the $U^{\uparrow,\downarrow}$ using the left/right eigenstates of the $H_{pt}^{\uparrow}$ and $H_{pt}^{\downarrow}$. These two PT symmetric Hamiltonians have the same eigenvalues, and we denote these two eigenvalues as E_1 and E_2, namely

$$E_1 = -\sqrt{\kappa^2-\Gamma^2},\ E_2 = -E_1 = \sqrt{\kappa^2-\Gamma^2}. \quad \text{(S3)}$$

For the left/right eigenstates, $H_{pt}^{\uparrow}$ and $H_{pt}^{\downarrow}$ are not equal to each other. By solving the function $H_{pt}^{\uparrow}|E^{\uparrow,R}\rangle = E|E^{\uparrow,R}\rangle$; $H_{pt}^{\downarrow}|E^{\downarrow,R}\rangle = E|E^{\downarrow,R}\rangle$ for right eigenstates and $\langle E^{\uparrow,L}|H_{pt}^{\uparrow} = \langle E^{\uparrow,L}|E$ and $\langle E^{\downarrow,L}|H_{pt}^{\downarrow} = \langle E^{\downarrow,L}|E$ for the left eigenvectors, we have

$$|E_1^{\uparrow,R}\rangle = \binom{-i\Gamma+E_1}{1},\ |E_2^{\uparrow,R}\rangle = \binom{-i\Gamma-E_1}{1}, \quad \text{(S4)}$$

$$|E_1^{\downarrow,R}\rangle = \binom{i\Gamma+E_1}{1},\ |E_2^{\downarrow,R}\rangle = \binom{i\Gamma-E_1}{1}. \quad \text{(S5)}$$

For the left eigenvectors, we have

$$\langle E_1^{\uparrow,L}| = (-i\Gamma+E_1, 1),\ \langle E_2^{\uparrow,L}| = (-i\Gamma-E_1, 1), \quad \text{(S6)}$$

$$\langle E_1^{\downarrow,L}| = (i\Gamma + E_1, 1),\ \langle E_2^{\downarrow,L}| = (i\Gamma - E_1, 1).\ \text{(S7)}$$

Using these eigenstates, we can expand the unitary operator in the eigen basis. For example, for the evolution operator of the up PT Hamiltonian, we have

$$e^{-i\,H_{pt}^{\uparrow}\tau_\uparrow} = e^{-i\,E_1\tau_\uparrow}\frac{|E_1^{\uparrow,R}\rangle\langle E_1^{\uparrow,L}|}{\langle E_1^{\uparrow,L}|E_1^{\uparrow,R}\rangle} + e^{-i\,E_2\tau_\uparrow}\frac{|E_2^{\uparrow,R}\rangle\langle E_2^{\uparrow,L}|}{\langle E_2^{\uparrow,L}|E_2^{\uparrow,R}\rangle}.\ \text{(S8)}$$

So, the propagator $U^{\uparrow,\downarrow}$ for one period time T reads

$$U^{\uparrow,\downarrow} = e^{-i\,H_{pt}^{\downarrow}\tau_\uparrow}\,e^{-i\,H_{pt}^{\uparrow}\tau_\uparrow}.\ \text{(S9)}$$

We present the light distribution versus the propagation distance for different length of T in the waveguide array using the beam propagation approach in Fig. **S3**.

The special case we consider is when the evolution time in the up and down PT Hamiltonians are equal, and the evolution time is determined by the eigenvalues of the PT Hamiltonian, namely:

$$\frac{T}{2} = \tau_\uparrow = \tau_\downarrow = \frac{\pi}{2\sqrt{\kappa^2 - \Gamma^2}}.\ \text{(S10)}$$

When this condition is satisfied, the propagator $U^{\uparrow,\downarrow}$ can be largely simplified, which leads to:

$$U^{\uparrow,\downarrow}(T) = \frac{1}{-\kappa^2+\Gamma^2}\begin{pmatrix}\kappa^2+\Gamma^2 & 2i\Gamma\kappa \\ -2i\Gamma\kappa & \kappa^2+\Gamma^2\end{pmatrix}.\ \text{(S11)}$$

There are two eigenstates for the $U^{\uparrow,\downarrow}(T)$, which are the magnification mode and decay model we used in the paper, namely

$$U^{\uparrow,\downarrow}(T)\begin{pmatrix}\frac{1}{\sqrt{2}} \\ -\frac{i}{\sqrt{2}}\end{pmatrix} = \frac{\kappa+\Gamma}{-\kappa+\Gamma}\begin{pmatrix}\frac{1}{\sqrt{2}} \\ -\frac{i}{\sqrt{2}}\end{pmatrix}, \left|\frac{\kappa+\Gamma}{-\kappa+\Gamma}\right| > 1.\ \text{(S12)}$$

And

$$U^{\uparrow,\downarrow}(T)\begin{pmatrix}-\frac{i}{\sqrt{2}} \\ \frac{1}{\sqrt{2}}\end{pmatrix} = \frac{-\kappa+\Gamma}{\kappa+\Gamma}\begin{pmatrix}-\frac{i}{\sqrt{2}} \\ \frac{1}{\sqrt{2}}\end{pmatrix}, \left|\frac{-\kappa+\Gamma}{\kappa+\Gamma}\right| < 1.\ \text{(S13)}$$

For effective Hamiltonian for the Floquet PT symmetric system, we use the relation that

$$e^{-iH_{eff}T} = U^{\uparrow,\downarrow}(T).\ \text{(S14)}$$

This Hamiltonian is used for the plot of the effective spectrum of the PT symmetric superlattice in the main text. In general, for H_{eff} we have:

$$H_{eff} = i\log\left[U^{\uparrow,\downarrow}(T)\right]/T \quad \text{(S15)}$$

To calculate the Logarithm of a matrix, the first step is checking if this matrix is a diagonalizable matrix. As we have shown, these are two eigenmodes of the matrix$U^{\uparrow,\downarrow}(T)$. So in the space of *V*, in which each column of *V* is an eigenvector of$U^{\uparrow,\downarrow}(T)$, we have $A = V^{-1}U^{\uparrow,\downarrow}(T)V$, where *A* is a diagonal matrix whose diagonal elements are eigenvalues of $U^{\uparrow,\downarrow}(T)$ and V^{-1} is the inverse matrix. Then $U^{\uparrow,\downarrow}(T)$ is a diagonalizable matrix, and the H_{eff} can be directly obtained. Hence, we have

$$H_{eff} = \frac{i\log\left[U^{\uparrow,\downarrow}(T)\right]}{T} = \frac{iV\log(A)\,V^{-1}}{T}$$
$$= \begin{pmatrix} 0 & \dfrac{i\sqrt{\Gamma^2-\kappa^2}\log[(\Gamma-\kappa)/(\Gamma+\kappa)]}{\pi} \\ \dfrac{-i\sqrt{\Gamma^2-\kappa^2}\log[(\Gamma-\kappa)/(\Gamma+\kappa)]}{\pi} & 0 \end{pmatrix}$$

(S16)

This is the effective Hamiltonian for the Floquet PT symmetric system for the special Floquet periodicity given in Eq. (S10), which is a non-Hermitian matrix when $\Gamma < \kappa$. The eigenvectors of H_{eff} is the same as the $U^{\uparrow,\downarrow}(T)$. For its eigenvalues we have

$$E_1^{H_{eff}} = \frac{-\sqrt{\Gamma^2-\kappa^2}\log[(\Gamma-\kappa)/(\Gamma+\kappa)]}{\pi},\ E_2^{H_{eff}} = \frac{\sqrt{\Gamma^2-\kappa^2}\log[(\Gamma-\kappa)/(\Gamma+\kappa)]}{\pi}. \quad \text{(S17)}$$

The corresponding eigenvectors are $\begin{pmatrix} 1/\sqrt{2} \\ -i/\sqrt{2} \end{pmatrix}$ and $\begin{pmatrix} -i/\sqrt{2} \\ 1/\sqrt{2} \end{pmatrix}$, which are the amplification $|\xi_{\mathrm{am}}\rangle$ and suppression $|\xi_{\mathrm{su}}\rangle$ modes separately. This is the detailed deviation for the results used in the main text.

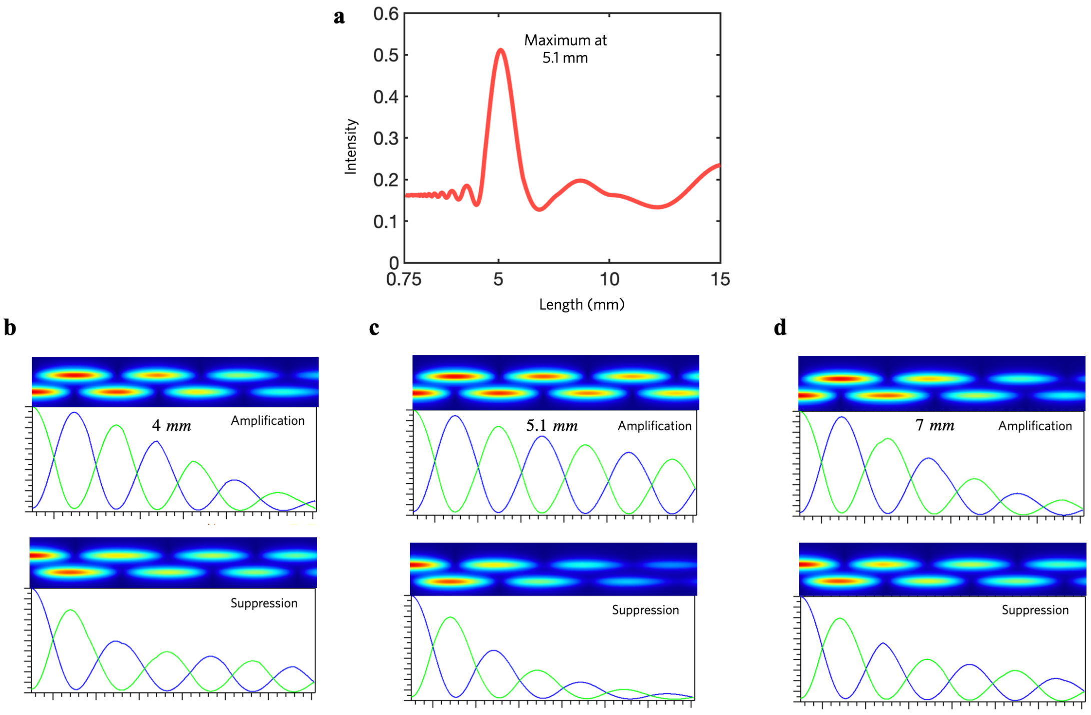

Fig. S3. Light evolution simulation with beam propagation method for different length of Floquet periodicity. Here we set the total length of the superlattice as 30 mm. We change the length of curved waveguide from 0.75 mm to 15 mm. The total light intensity in the end of the waveguide array is measured. a, The light intensity versus the length of the period. As shown in the figure, the output light intensity reaches maximum at 5.1 mm, which is the length for the maximum signal amplification in Floquet Parity-Time symmetric photonics. **b-d,** The light distribution versus the propagation distance for the length of 4 mm (smaller than the length of the maximum signal amplification), for the length at the maximum signal amplification (5.1 mm), and for the length of 7 mm (bigger) than the length of the maximum signal amplification) As shown in the figures, when the Floquet periodicity is at 5.1 mm, the light intensity is stronger for the amplification mode compared with the 4 mm case and 7 mm. The light intensity also reaches minimum when length is 5.1 mm for the suppression mode. This point achieves both the maximum light amplification and maximum light suppression depending on different initial states.

S4. Additional functionalities of Floquet PT-symmetric systems

In this section, we present the enhanced capabilities of our Floquet PT-symmetric system. We demonstrate that our system facilitates various modifications to the propagation of light, including power control based on system parameters such as gain and loss magnitude, manipulation of different initial states, including their phases, as well as mode selection of the input signal, and other potential functionalities.

We start with different initial states, where the initial light intensity remains consistent across different states, with the only distinction being their respective phases. This characteristic is crucial for the Floquet PT-symmetric system's sensitivity and responsiveness to phase variations, even in the presence of constant light intensity initial conditions. By launching the light with diverse initial phases into our Floquet PT-symmetric system and recording the power at the output facet, we establish a linear correlation between the phase of the initial states and the output power of the light. This correlation is shown in Fig. S4, where panel (a) demonstrates the straightforward manipulation of output power through phase control. Furthermore, this capability for power control extends to the selection of modes for the initial states, as exemplified by the transition from a mixed mode to a distinct amplification mode, elucidated in panels (b) through (g).

In addition to phase control, our system exhibits a remarkable capacity to modulate light intensity through the manipulation of gain and loss magnitudes introduced in the waveguides. By manipulating these parameters, we can significantly influence the extent of power modification, as demonstrated in Fig. 5S. Notably, we observe that higher values of Γ lead to a wider range of power modification, while smaller Γ values enable more precise control over light intensity. The visualization of light distribution within the waveguide array, as illustrated in panels (b)-(d) of Fig. 5S, provides further support and enhances our understanding of the system's behavior under different conditions. This highlights the system's ability to exert manipulation over light propagation by choosing different system parameters. Our Floquet PT-symmetric system enables a large regime of power control with a large gain/loss magnitude or a small regime but more accurate justification with a small gain/loss magnitude.

By integrating these functionalities, our Floquet PT-symmetric system emerges as a promising tool in photonics, exhibiting remarkable proficiency in precisely manipulating the propagation of light for a diverse range of applications. The system's ability for governing light behavior based on the initial phase, coupled with its modulation intensity through meticulous control of gain and loss magnitudes, not only advances scientific advancements but also fosters the emergence of groundbreaking applications, thereby significantly enriching the field.

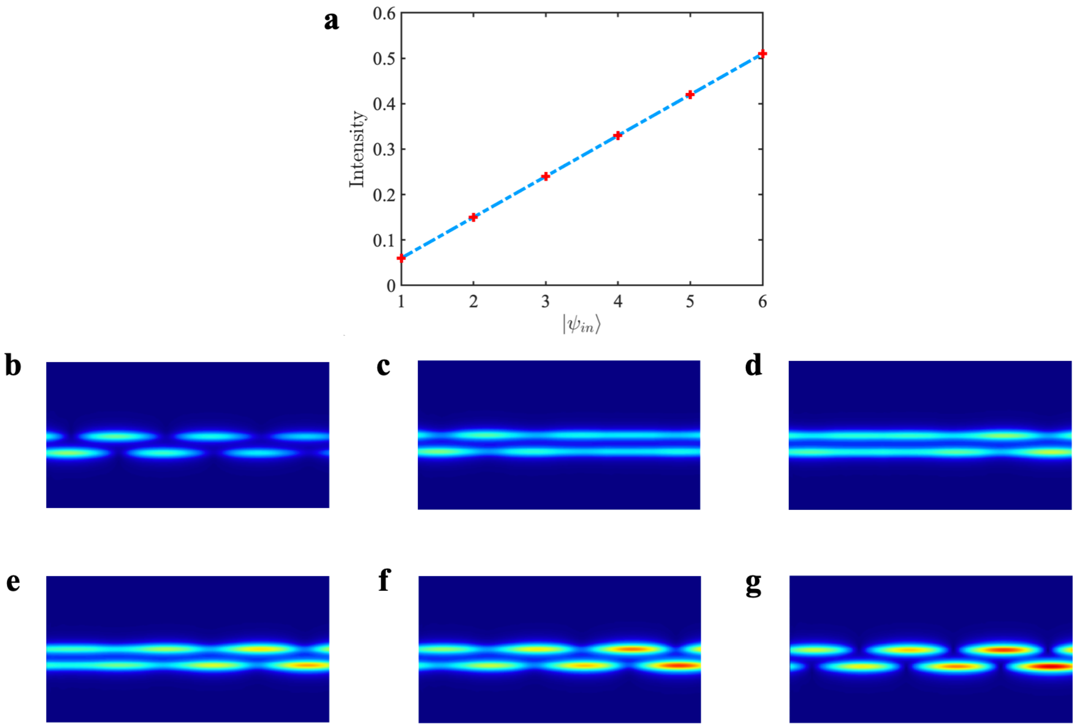

Fig. S4. Controlling the output signal using the different phases of the initial states. Here we plot the output light intensity for different initial states. In the simulation, all the initial states have the same light intensity at the beginning and the only difference between them is their phase, namely. We let the light propagate in our Floquet PT-symmetric structure and monitor the output power for different initial states. As shown in the figure, by choosing the phase of the initial states, we achieve a linear control of the output power for the injected light (a). The detailed evolution of the light is shown in (b)-(g). From these figures, we also see our structure enables the mode selection for the initial states. We see the output mode

in the system change from a mixed mode to a clear amplification mode. These results show the novel functionality introduced by our Floquet structure.

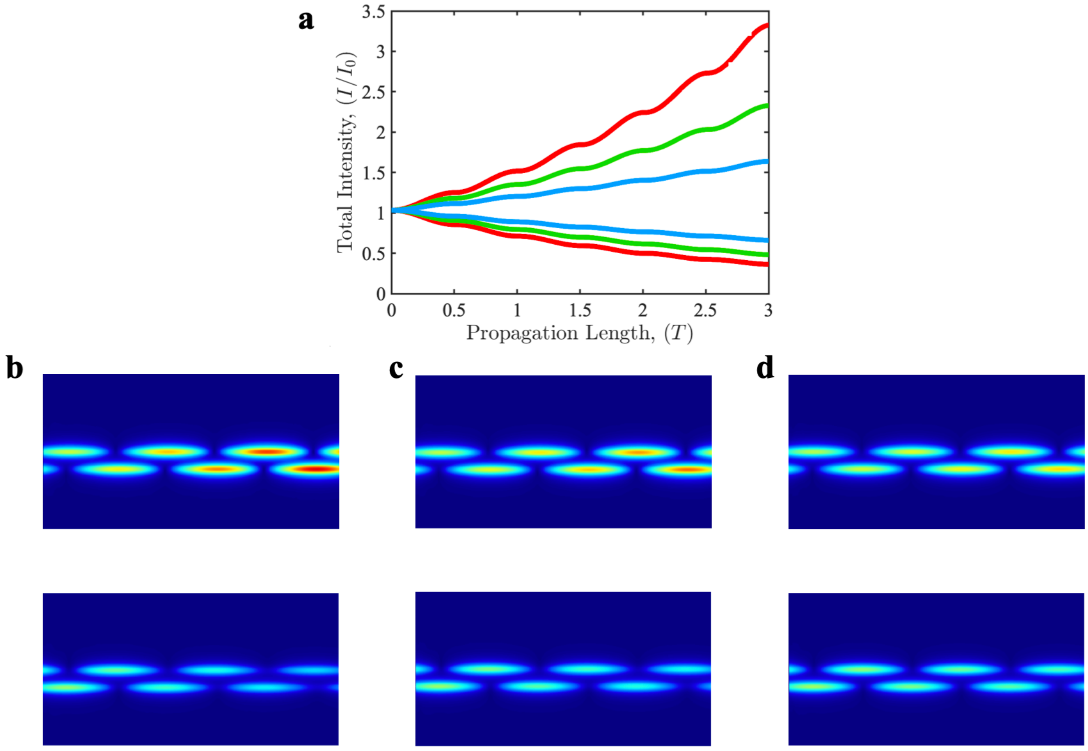

Fig. 5S. The total light intensity versus the propagation distance with different gain/loss magnitudes. In the simulation, the Γ = 20 m^{-1}(blue lines); Γ = 30 m^{-1}(green lines); Γ = 50 m^{-1}(red lines) and the coupling κ = 314 m^{-1}. In (a) we plot the total light intensity versus the propagation distance for the amplification mode and suppression modes. As shown in the figure, as we increase the Γ, the modification range of the output power becomes larger. On the other hand, with a small value of Γ, we can achieve a more accurate justification of the power together with the results in Fig. 4S. The light distributions in the waveguide array for different gain/loss magnitudes are shown in (b)-(d). The upper panel presents the light evolution for the amplification modes for different gain/loss strengths and the lower panel is the corresponding suppression mode. Their total light intensity is shown in (a), which determines the maximum and minimum output power from the chip.
